# Shared Mechanisms in the Estimation of Self-Generated Actions and the Prediction of Other’s Actions by Humans

**DOI:** 10.1523/ENEURO.0341-17.2017

**Published:** 2017-01-13

**Authors:** Tsuyoshi Ikegami, Gowrishankar Ganesh

**Affiliations:** 1 Center for Information and Neural Networks, National Institute of Information and Communications Technology, 1-4 Yamadaoka, Suita City, Osaka 565-0871, Japan; 2 CNRS-AIST JRL (Joint Robotics Laboratory), UMI3218/CRT, Intelligent Systems Research Institute, National Institute of Advanced Industrial Science and Technology (AIST), Tsukuba Central 2, 1-1-1 Umezono, Tsukuba, Ibaraki 305-8568, Japan; 3 Brain Information Communication Research Laboratory Group, ATR, 2-2-2 Hikaridai, Kyoto 619-0288, Japan Seika-Cho, Soraku-gun

**Keywords:** Action observation, action prediction, forward model, motor system

## Abstract

The question of how humans predict outcomes of observed motor actions by others is a fundamental problem in cognitive and social neuroscience. Previous theoretical studies have suggested that the brain uses parts of the forward model (used to estimate sensory outcomes of self-generated actions) to predict outcomes of observed actions. However, this hypothesis has remained controversial due to the lack of direct experimental evidence. To address this issue, we analyzed the behavior of darts experts in an understanding learning paradigm and utilized computational modeling to examine how outcome prediction of observed actions affected the participants’ ability to estimate their own actions. We recruited darts experts because sports experts are known to have an accurate outcome estimation of their own actions as well as prediction of actions observed in others. We first show that learning to predict the outcomes of observed dart throws deteriorates an expert’s abilities to both produce his own darts actions and estimate the outcome of his own throws (or self-estimation). Next, we introduce a state-space model to explain the trial-by-trial changes in the darts performance and self-estimation through our experiment. The model-based analysis reveals that the change in an expert’s self-estimation is explained only by considering a change in the individual’s forward model, showing that an improvement in an expert’s ability to predict outcomes of observed actions affects the individual’s forward model. These results suggest that parts of the same forward model are utilized in humans to both estimate outcomes of self-generated actions and predict outcomes of observed actions.

## Significance Statement

Do the neural circuits referred to as forward models, which help humans estimate sensory outcomes of self-generated actions, also help them predict the outcome of other’s actions? To address this question, we examined the interactions between one’s estimation of self-generated actions and the prediction of other’s actions. We first show that learning to predict the outcome of observed actions affects one’s ability to both produce actions and estimate the outcome of self-generated actions. Next, using a model-based analysis, we show that these affects cannot be explained without a change in one’s forward model. Our results suggest the presence of shared mechanisms in the human brain for the estimation of self-generated actions and the prediction of other’s actions.

## Introduction

The ability to predict outcomes of observed actions performed by others is fundamental to the human ability to interact physically (Ganesh et al., 2014; Takagi et al., 2017) and socially (Frith and Frith, 1999; Pickering and Garrod, 2013) with each other. Previous motor studies in humans (Wolpert et al., 1995; Desmurget and Grafton, 2000; Christensen et al., 2007; Miall et al., 2007), nonhuman primates (Duhamel et al., 1992; Sommer and Wurtz, 2002; Mulliken et al., 2008), birds (Troyer and Doupe, 2000), and insects (Mischiati et al., 2015) have shown that the sensory consequences of self-generated actions are estimated by neural circuits in the motor system, referred to as forward models (Wolpert and Kawato, 1998; Shadmehr and Wise, 2005). However, whether the same forward models are involved in the prediction of observed actions remains controversial (Clark, 2013; Zimmermann et al., 2013; Pickering and Clark, 2014).

Researchers against the idea argue that outcome prediction of observed actions can be achieved by learning the associations between previously observed actions and their outcomes (Jellema and Perrett, 2005; Mahon and Caramazza, 2008; Caramazza et al., 2014). They dismiss the requirement of a forward model for this purpose. On the other hand, researchers supporting the idea propose that humans may simulate the motor command corresponding to an observed action and then use their forward model (used to estimate self-generated actions) to improve the outcome prediction of the observed action (Wolpert et al., 2003; Oztop et al., 2005; Pickering and Garrod, 2013).

In our recent study, we used a novel understanding learning paradigm (Ikegami and Ganesh, 2014) with darts experts to show that a change in a darts expert’s ability to predict the outcome of dart throws made by another individual affects the expert’s own darts performance. While this result, and reports from other previous studies (Knoblich and Flach, 2001; Flanagan and Johansson, 2003; Falck-Ytter et al., 2006; Aglioti et al., 2008; Kanakogi and Itakura, 2011; Tidoni et al., 2013; Ikegami and Ganesh, 2014; Michael et al., 2014; Mulligan et al., 2016) suggest that the human motor system is involved in the prediction of observed actions, direct evidence for the involvement of the forward model in the outcome prediction process remains lacking.

To address this issue, here we analyzed the darts experts’ ability to estimate the outcome of their own actions (which we call self-estimation) using new data from our previous experiment (Ikegami and Ganesh, 2014). We first analyzed how an expert’s self-estimation changes when we induce a change in his ability to predict the outcome of observed dart throws (which we call outcome prediction). If a same forward model determines both self-estimation and outcome prediction, then we expected that a change in one may affect the other. To anticipate our results, we found this to be true: a change in the outcome prediction, induced by the understanding learning, results in a progressive deterioration in the expert’s self-estimation as well as darts performance. We then developed a state–space model to quantitatively explain the interaction between the changes in self-estimation and darts performance and the change in outcome prediction. Our model-based analysis showed that the deterioration in the self-estimation and darts performance by the experts are both quantitatively explained by a change in their forward model during outcome prediction, suggesting that the forward model is used for the outcome prediction of observed throws.

## Methods

### Subjects

Twenty-one male darts experts [players with a rank of A or above on the International online darts game scale (VSPHOENIX: http://gs.phoenixdart.com/vsphoenix/class.php); aged 22–48 years] took part in our study. We also included three novice dart throwers (three males, aged 30–35) and three novice bowlers (three males, aged 30–34; one person took part in our study as both a novice dart thrower and a novice bowler) as models whom the experts watched. The novice darts players were all individuals who threw darts for the first time. The novice bowlers were individuals who had bowled <5 times in their life. All experiments were conducted according to the principles in the Declaration of Helsinki. The subjects gave informed consent before the experiment, and the experiments were approved by the local ethics committee in National Institute of Information and Communications Technology.

We purposely chose to use experts as subjects in our experiment because sports experts are known to predict the outcome of their specialized actions very well, both when they observe others performing those actions (Aglioti et al., 2008; Yarrow et al., 2009; Urgesi et al., 2012) and when they perform themselves (Knoblich and Flach, 2001; Mulligan et al., 2016). More importantly, an expert will not explicitly imitate the observed action by the novice, which allows us to exclude a confounding factors of possible explicit strategies as a cause of our result (Maslovat et al., 2010).

### Videorecording of observed actions

**Figure 1.**
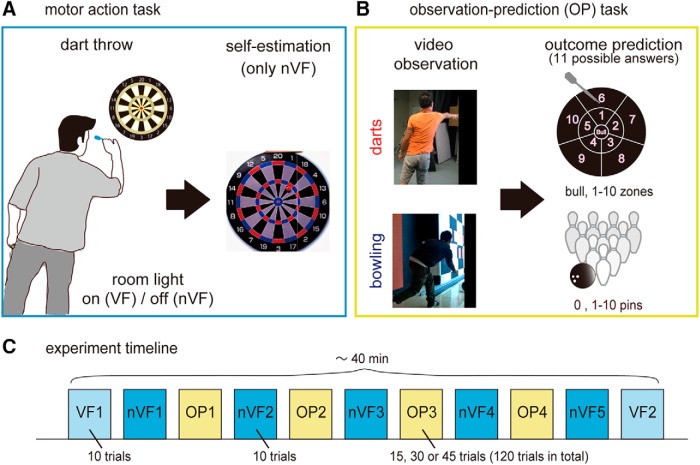
Experiment. ***A***, The experiment consisted of two motor action tasks, one in which the darts experts threw darts in the presence of visual feedback (VF; where their darts land on the dartboard) and second, in the absence of visual feedback (nVF). After every throw in the nVF condition, the darts experts were asked to self-estimate where their dart had landed on the board by placing a magnet on second dartboard placed behind them. ***B***, In the observation-prediction (OP) tasks, the darts experts watched the video of either a novice dart thrower or a ten-pin bowler (snapshots shown), made a prediction of the outcome of each throw, and were given the feedback of the correct outcome orally by the experimenter. The chance level for the OP tasks for both the bowling and darts observation sessions was 9.09% (1/11 × 100). Each experiment session followed the sequence of blocks as shown in ***C***).

The novice dart throwers and novice bowlers were videorecorded from behind (and right) of the novices. They made 36 throws each in which they aimed for either the dartboard center (in case of darts) or a strike (in case of bowling). The videorecorded throws from each novice were shuffled and used to create a series of 120 throws that were shown to the darts experts. Moreover, part of the video was masked such that the experts could see the kinematics of the novice actions and the ball/dart release but were not able to see the ball/dart trajectory, the outcome of dart landing positions on the board, or the number of bowling pins felled (snapshot of video in Fig. 1*B*).

### Experimental conditions

Each experiment in this study consisted of three tasks: dart throwing with no visual feedback (nVF), dart throwing with visual feedback (VF), and the observation–prediction (OP) task.

#### Darts throwing with no visual feedback (nVF)

The experts threw darts aiming for the center of a dartboard but were not allowed to see where the dart thrown by them landed on the board (Fig. 1*A*). This was achieved by switching off the room light during each throw, by use of a switch that the experts held between the palm and fingers in their left hand and operated while throwing the darts. They practiced using the switch before starting the experiment.

Each trial started with the room light on, which allowed the expert to take aim on the dartboard. The experts held the light switch in their closed left hand and were asked to simultaneously open their left hand when they released the dart with their right hand. Opening the hand switched off the room light. A customized experiment room with black curtains ensured that the room light off made the room so dark that the dart flight trajectory and dartboard were not visible to the subjects. They were asked to turn around immediately after they threw a dart and while the room light was still off. The light was then turned on and the expert was asked to self-estimate their performance outcome: mark the position he estimated his dart to have landed by placing a magnet on a magnetic dartboard (of size identical to the one they aimed for) pasted on the back wall of the room. During this period, an experimenter measured the dart-landing position of the thrown dart and removed it from the board. The experts then turned back, was provided with a new dart and went on to make the next throw. The experts got no feedback of where their throw landed. Each nVF block included 10 dart throws and the corresponding self-estimations. The dart-landing positions and self-estimated positions were measured in the *x-y* coordinate with the origin at the center of the board.

#### Darts throwing with full visual feedback (VF)

The experts made dart throws but the room light was kept on throughout the condition (Fig. 1*A*). However, to equalize the conditions, they were asked to operate the light switch as in the nVF condition, although this did not make any change in the lighting conditions. Each VF block included 10 dart throws and there was no self-estimation during the VF condition.

#### Novice action observation–prediction (OP)

The experts sat on a chair and watched the video of either a novice dart thrower or a novice bowler on the computer monitor in front of them (Fig. 1*B*). Each expert watched either the bowling or darts video from a single novice throughout any one experimental session. After each throw video (either darts or bowling), the expert was asked to predict the outcome of the novice action. They were instructed to write down the prediction on a sheet of paper provided to them. In the bowling observation session, there were 11 possible outcomes from 0 pins to 10 (strike). In the darts observation session, to equalize the difficulty, we divided the dartboard into 11 parts as well (see Fig. 1*B*). There was no time limit on the OP task. Once the experts announced that they had written their prediction, they were provided with the correct answer orally by an experimenter which they were asked to write besides their prediction. After this, they went on to watch the video of the next throw. Each OP block included 15, 30, or 45 throws to watch. In total, the experts watched 120 trials through four OP blocks in each experimental session.

### Experimental procedure

We conducted three experiments as reported in our previous study (Ikegami and Ganesh, 2014). However, to address the new question of this study, this report shows and analyzes data from two of the experiments.

### Practice session

In the two experiments, the experts started with a practice period. First, the experts were allowed to take their time and throw darts to acclimatize themselves to our experimental environment. This was followed by instructions on the use of the light switch. Then, the experts again practiced their throws till the light switch was not believed to interfere with their concentration. All experts felt comfortable with the switch with a practice of <15 min.

### Experiment session

In each experiment session, the experts were required to throw 70 darts (aimed for the center of the dartboard) over a VF block, followed by five nVF blocks that were interleaved with four OP blocks, and finally ending with a second VF block (see Fig. 1*C*).

Each experiment had two experimental sessions. In each session and in both the experiments, the order of blocks remained as mentioned above with changes only in the OP tasks [either in the observed video (darts/bowling) or in the instructions]. The two experimental sessions on the same day were separated by a 20-min break, which was followed by a practice session similar to that at the beginning of the first session.

### Experiment 1

Experiment 1 involved 16 dart experts and extended over 2 d, with two sessions on each day. Half of the experts had a darts observation session followed by a bowling observation session on the first day, and then *vice versa* on the second day. The other half had the opposite order of sessions on each day. In the beginning of each session, the experts were instructed that “the novice in the video aims for the center of the board [when it was a darts observation session] or a strike [when it was a bowling observation session].” Furthermore, the experts were provided with the feedback of the correct answers in all the OP blocks. The darts and bowling observation sessions in Experiment 1 were therefore trainable observation sessions, because the instruction and feedback provided by us allowed the experts to improve their ability to predict the outcome of the novice’s actions (see Fig. 3).

The two experiment days for any expert were separated by at least 4 d. Note that data from only the first session of each day is analyzed in this article. The data form the second session on each day of Experiment 1 were collected to investigate interference effects that are not discussed here.

### Experiment 2

Experiment 2 involved 16 subjects (11 of whom had participated in Experiment 1; the other 5 subjects were new recruits) in an extended single session. Experiment 2 used only darts observation sessions for the OP task, and each expert watched the video of a different novice individual than he had observed in Experiment 1. Experiment 2 differed from Experiment 1 in two critical aspects. First, although the experts in Experiment 2 watched the darts video of a novice trying to hit the center (same as in Experiment 1), they were clearly instructed at the start of the experiment with a lie that “the novice in the video does not always aim for the center but aims for unknown targets provided by us, and we display only those trials in which they were successful.” This misinformation was expected to alter the experts’ beliefs about the novice’s behavioral goal. Second, the experts were not provided with any feedback of the correct answers after each prediction in the OP blocks. These two differences helped us suppress the two prediction error feedbacks available to the experts to improve their prediction ability (Ikegami and Ganesh, 2014). The first is the kinematics prediction error: the difference between the novice kinematics actually observed and the kinematics expected by the expert based on the goal he believed the novice was aiming for (removed due to goal misinformation in Experiment 2). Second is the outcome prediction error: the difference between the outcome predicted by the expert from the observed novice action and the actual outcome provided to the expert orally by the experimenter (removed in Experiment 2). In contrast to the trainable observation sessions of Experiment 1, the suppression of these two types of prediction errors inhibited the experts from improving the outcome prediction of the novice’s actions (see Fig. 3). We therefore refer to the darts observation session of Experiment 2 as untrainable observation sessions.

### Experimental data analysis

#### Darts performance and self-estimation performance

**Figure 2.**
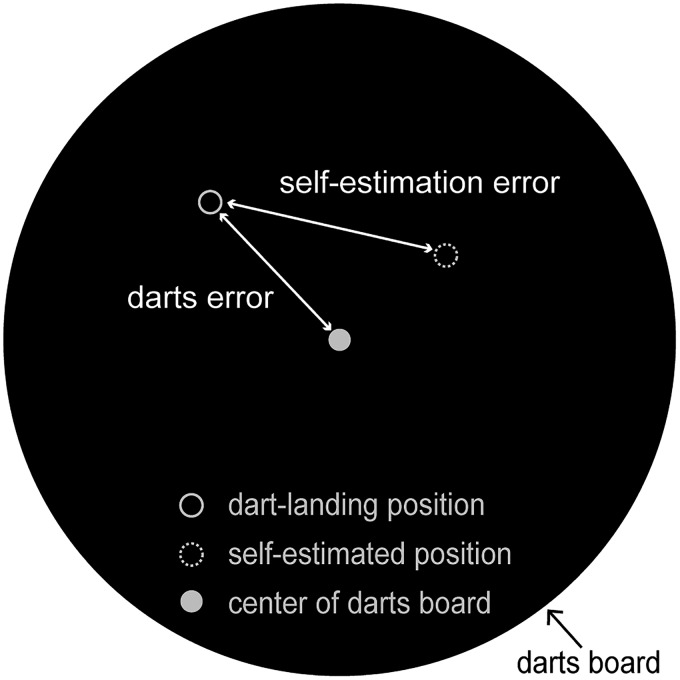
Darts performance measures and nomenclature. The darts error of each throw in the VF and nVF blocks was defined as the unsigned distance of the dart-landing position (solid-outlined circle) from the board center (closed circle). The self-estimation error of each throw in the nVF blocks was defined as the unsigned distance between the dart-landing position and the self-estimated position (dotted circle).

**Figure 3.**
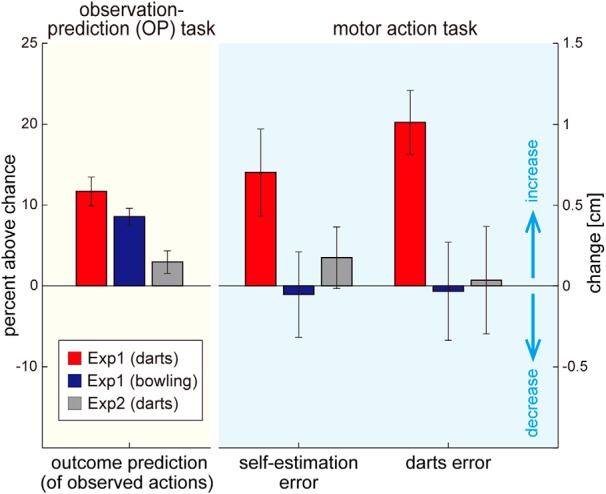
Relation between observation prediction and motor action. The darts experts’ outcome prediction of observed darts actions (left red bar) and bowling actions (left blue bar) improved in the trainable observation sessions (Experiment 1). The outcome prediction of observed darts actions did not improve in the untrainable observation session (Experiment 2), although between them the experts watched dart actions by the same novice throwers in Experiments 1 and 2. The experts’ self-estimation error and darts error increased only when they watched the novice’s darts actions and their outcome prediction improved (Exp. 1, middle and right red bars) but not when they watched bowling actions (Exp. 1, middle and right blue bars) or when they failed to improve their outcome prediction of observed darts actions (Exp. 2, middle and right gray bars). Error bars indicate standard error.

The darts error by a subject was defined as the unsigned distance of their dart-landing position from the board center in nVF or VF blocks (see Fig. 2). The change in the darts error between the two VF blocks was used to evaluate the dart performance as in our previous study (Ikegami and Ganesh, 2014) and is plotted in the right (blue) panel of Fig. 3. To avoid effects of initial anxiety, the darts error in the first VF block was evaluated as the average only over the last three throws of the VF. On the other hand, the darts error in the second VF block was evaluated as the average over the first five throws to minimize artifacts due to the trial-by-trial feedback corrections performed by the experts in the presence of the visual feedback (Ikegami and Ganesh, 2014).

Likewise, self-estimation error of a subject was then defined as the unsigned distance between the dart-landing position and the self-estimated position in nVF blocks (see Fig. 2). The change in the self-estimation error between nVF1 and nVF5 blocks was used to evaluate the self-estimation performance and is plotted in the right (blue) panel of Fig. 3. The self-estimation error in each nVF block was evaluated as the average over all the 10 throws.

**Figure 4.**
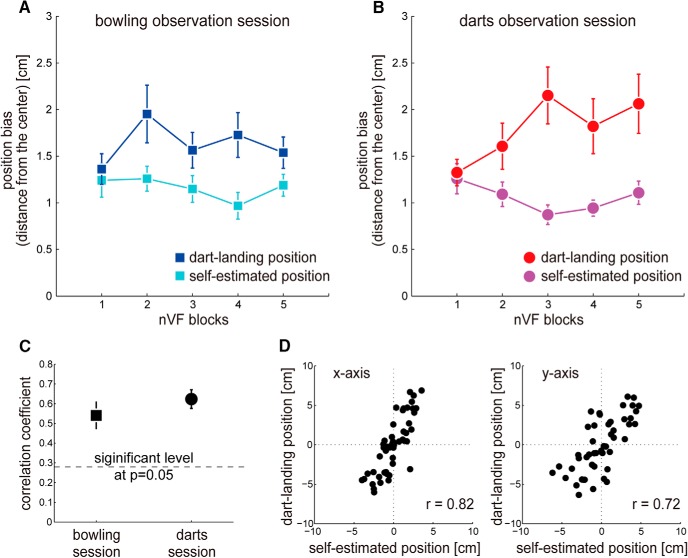
Experimental results of behavioral trends. Increase in dart-landing position bias: The dart-landing position deviated progressively from the board center after outcome prediction of observed darts actions (B, red trace) but not after outcome prediction of bowling actions (***A***, blue trace) in Experiment 1. Constant self-estimated position bias: The self-estimated position did not change through the experiment, both after outcome prediction of darts actions (***B***, magenta trace) and after outcome prediction of bowling actions (***A***, cyan trace). Correlation between dart-landing and self-estimated positions: However, a substantial correlation was observed between dart-landing and their self-estimated positions in both darts (***C***, ***D***) and bowling (***C***) observation sessions. Individual data from a representative darts expert in the dart observation session is shown in ***D***, and the correlation coefficient averaged over the *x*- and y-axis across all darts experts is shown in ***C***. The dashed line in ***C*** represents the significance level at *p* = 0.05. Error bars indicate standard error.

Next, to closely examine how the outcome prediction of observed actions affects the change of the darts error and the self-estimation error in the trainable observation sessions (Experiment 1), we analyzed changes in dart-landing position and self-estimated position in terms of their variance and position bias during the nVF condition. To evaluate changes in the variance, we calculated the 2D standard deviations ($\sigma_{xy}=\sqrt{\frac{\sigma_{x}^{2}}{2}\frac{+\sigma_{y}^{2}}{2}}$, where σ represents standard deviation) in each nVF block (10 trials) of dart-landing and self-estimated positions. To evaluate the changes in the position bias, we calculated the distance of the averaged position of dart-landing or self-estimated positions in each nVF block from the board center; they are plotted in Fig. 4*A*, *B*.

Finally, to examine the relationship between individual dart-landing and self-estimated positions in the trainable observation sessions of Experiment 1, we performed a Pearson’s correlation analysis for each axis using the data from all the nVF conditions (50 data points). Because we found a significant increase only in the dart-landing position bias across nVF blocks in the darts observation session (Fig. 4), we corrected the nonstationarity before calculating the correlation; the correlation coefficient of the dart-landing position in the darts observation session was calculated for each *x* and *y* dimension after subtracting the averaged position in each nVF block from the original position in each trial. The other data (dart-landing position in the bowling observation session and self-estimated positions in both darts and bowling observation sessions) were not corrected because no nonstationarity was observed (Fig. 4). A representative data from an individual is plotted in Fig. 4*D*. For the group analysis, we averaged the correlation coefficients over the *x* and *y* dimensions to obtain the averaged correlation coefficient (plotted in Fig. 4*C*) and performed a one-sample *t* test to examine whether the correlation coefficient was significantly different from *r* = 0.279 (significance level at *p* = 0.05 for 50 data points).

#### Outcome prediction performance

The OP tasks in both the darts and bowling observation sessions required the experts to predict from one of 11 possible outcomes in each trial. A prediction was deemed successful only if the darts zone or the bowling pins felled matched the correct outcome. Therefore, the chance level for both OP tasks was 1/11 × 100 = 9.09%. The outcome–prediction change was evaluated as the percentage of total correct predictions above chance, as in our previous study (Ikegami and Ganesh, 2014).

The OP change was averaged across the experts for each experiment and is plotted in the left (yellow) panel of Fig. 3. One expert was excluded from the analysis of the OP task in Experiment 2 because he missed writing down his predictions in some trials, leading to a mismatch between the presented videos and his answers.

For all reported values, we report the mean and standard deviation in the article. On the other hand, error bars in the figures represent standard error.

### Model-based analysis

**Figure 5.**
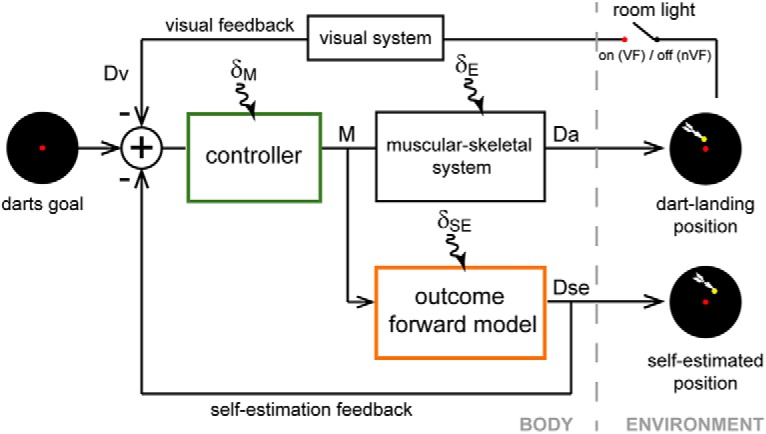
Proposed model. The model assumes that given a darts goal, a darts expert utilizes his controller to plan an appropriate motor command **M**, which is then fed to the musculoskeletal system to execute the required dart throw. The error in the throw is observed by the visual system when the room light is on and is used to correct the action in the next trial. The model assumes that in addition, the motor command **M** can be used to self-estimate the outcome of a throw using the outcome forward model, the output from which is used to correct the subsequent throw even when the room light is off. The model assumes the controller, the outcome forward model, and the muscular skeletal system to be affected by planning noise (**δ_M_**), self-estimation noise (**δ_SE_**), and execution noise (**δ_E_**), respectively.

The popular structure of the motor system assumes it to consist of two key components, the forward model and the controller (Wolpert, 1997; Pickering and Clark, 2014; Fig. 5). In our experiments, we observed that the improvement in the darts experts’ abilities to predict novice actions affected their own self-estimation as well as darts performance. To understand what changes in the outcome forward model and controller contribute to these effects, we used a state–space model (Thoroughman and Shadmehr, 2000; Shadmehr and Wise, 2005; Smith et al., 2006) to simulate the experts’ behavior in the trainable observation sessions (Experiment 1). The model assumes that the motor plan to execute a dart throw is developed using the darts controller and sent to the musculo-skeletal system to produce the action. The same motor plan can be used to self-estimate the outcome of the dart throw using the outcome forward model. Note that the popular terminology of forward model (Shadmehr and Wise, 2005; Diedrichsen et al., 2010; Friston, 2011) usually refers to the mapping between individual motor commands and sensory consequences during individual movements. This mapping is believed to be essential for online feedback control in the presence of neural delays and noises (Todorov and Jordan, 2002; Todorov, 2004). On the other hand, our model structure aims to explain the trial-by-trial variations (based on the outcome feedback) in the experts’ throws, similar to many reaching adaptation studies (Thoroughman and Shadmehr, 2000; Donchin et al., 2003; van Beers, 2009; Yokoi et al., 2011). We therefore prefix outcome in our nomenclature of the forward model in Fig. 5 because it represents a forward mapping between an action and its outcome on the environment (Miall, 2003)—specifically, between the throw kinematics and the consequent dart-landing position on the dartboard. The traditional forward models for online control of individual movements (Miall and Wolpert, 1996) would be encapsulated within the controller of Fig. 5.

#### Model of the expert darts performance and self-estimation performance

The trial by trial changes in the motor plan, self-estimation and dart throw were modeled as follows:

(1) $$M(i)=M(i-1)+\gamma \cdot D_{se}(i-1)+\delta_{M}\left[\begin{array}{c} \varepsilon^{x}\\ \varepsilon^{y} \end{array}\right],$$

(2) $$D_{se}(i)=M(i)+\delta_{SE}\left[\begin{array}{c} \eta^{x}\\ \eta^{y} \end{array}\right],$$

(3) $$D_{a}(i)=M(i)+\delta_{E}\left[\begin{array}{c} \xi^{x}\\ \xi^{y} \end{array}\right].$$

Vectors and matrices are written in bold, and scalars are represented in italics. Eq. 1 describes a trial-by-trial motor planning through the controller (see Fig. 5). Our model assumes that for an intended darts goal, a dart expert plans the required motor command $M=\left[\begin{array}{c} m^{x}\\ m^{y} \end{array}\right]$ in the *i* th trial. Here, *m^x^* and *m^y^* represent the internal state specifying the *x* and *y* position of thrown darts on the dartboard, respectively. The model assumes that in the absence of the visual feedback **D_v_** (available only in the VF conditions when the room light is on), the self-estimated position **D_se_** is used as feedback to modify the throws over trials. $\gamma =\left[\begin{array}{cc} \gamma^{x} & 0\\ 0 & \gamma^{y} \end{array}\right]$ represents the feedback gain. Furthermore, the motor command **M** is affected by planning noise with a standard deviation of $\delta_{M}=\left[\begin{array}{cc} \delta_{m}^{x} & 0\\ 0 & \delta_{m}^{y} \end{array}\right]$. ε^*x*^ and ε^*y*^ are Gaussian random in time with mean 0 and variance 1. Eq. 2 describes the self-estimation by the outcome forward model (see Fig. 5), which transforms the motor command to the estimated action outcome in terms of the self-estimated position **D_se_** in the *i* th trial. The self-estimation is affected by the estimation noise with a standard deviation of $\delta_{SE}=\left[\begin{array}{cc} \delta_{se}^{x} & 0\\ 0 & \delta_{se}^{y} \end{array}\right]$. η^*x*^ and η^*y*^ are Gaussian random in time with mean 0 and variance 1. Finally, Eq. 3 describes the motor execution that transforms from the motor commands to the musculoskeletal consequence in terms of the dart-landing position **D_a_** in the *i* th trial. The motor execution is affected by the execution noise with a standard deviation of $\delta_{E}=\left[\begin{array}{cc} \delta_{e}^{x} & 0\\ 0 & \delta_{e}^{y} \end{array}\right]$. ξ^*x*^ and ξ^*y*^ are Gaussian random in time with mean 0 and variance 1.

#### Simulation of the nVF behavior during the bowling observation session

With Eqs. 1–3, we first simulated the expert darts performance and self-estimation performance in the bowling observation session (Extended Data 2). As these did not change through the session (blue data in Fig. 3, right panel), we assumed that the motor system was unaffected by the observation of the novice actions in the session. We defined two noise ratios. First, the ratio between the self-estimation noise and execution noises was defined as $w_{1}=\left[\begin{array}{cc} w_{1}^{x} & 0\\ 0 & w_{1}^{y} \end{array}\right]$. Because the sum of self-estimation noise and execution noise can be estimated by the subtraction of **D_se_** (Eq. 2) from **D_a_** (Eq. 3), we get ${\left|\delta_{E}\right|}^{2}=Var(D_{a}-D_{se})\cdot W_{1}$ and ${\left|\delta_{SE}\right|}^{2}=Var(D_{a}-D_{se})\cdot (I-W_{1})$. Second, the ratio between the planning noise and execution noise was defined as $w_{2}=\left[\begin{array}{cc} w_{2}^{x} & 0\\ 0 & w_{2}^{y} \end{array}\right]$ as in previous studies (van Beers, 2009; van Beers et al., 2013) such that $\delta_{M}=W_{2}\cdot \delta_{E}$.

The model thus has three free parameters, **w_1_**, **w_2_**, and ***γ***. In fact, as both the self-estimation and darts performance remain essentially constant through the bowling observation sessions (Fig. 4*A*), multiple parameter sets (with the constraint of −1 < γ < 0) can reproduce the dart-landing and self-estimated positions. However, the degree of correlation between the dart-landing and self-estimated positions is determined by a balance between these parameters. We start with a naive assumption that the variances of self-estimation, execution, and planning are equal, resulting in $w_{1}^{x}=w_{1}^{y}=0.5$ and $w_{2}^{x}=w_{2}^{y}=1$. These values could reproduce the strong correlations (*r* > 0.6) observed between the dart-landing and self-estimated positions in the experimental data—again, provided that −1 < γ < 0. We then selected a median learning gain in this range: γ^*x*^ = γ^*y*^ = −0.5. We later show that this choice of parameters does not affect our conclusions (see Results and Fig. 7). We also discuss the parameter selection again in the Discussion.

**Figure 6.**
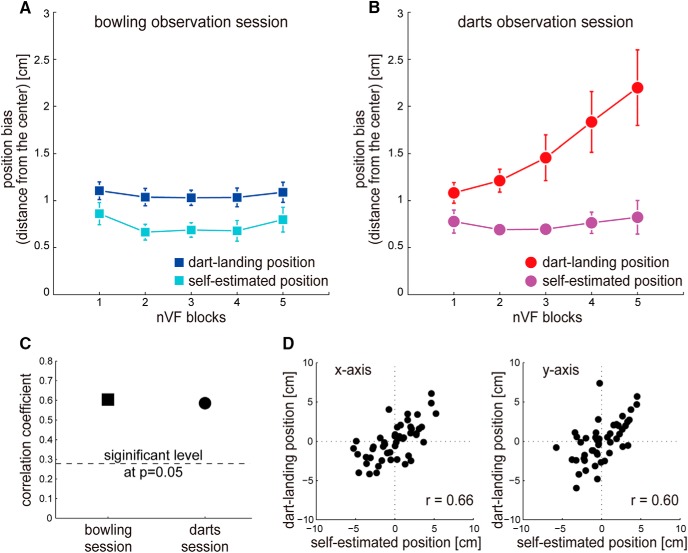
Simulation results for behavioral trends. Compare ***A–D*** with Fig. 4*A–D*, which uses the same plots of actual experiment data. The simulations using the *C^×^F^+^* model can reproduce the evolution of the experts’ behavior: dart-landing position (blue and red traces in ***A*** and ***B***, respectively), self-estimated position (cyan and magenta traces in ***A*** and ***B***, respectively), and the substantial correlations between these two positions (***C***) in both the bowling (square) and darts (circle) observation sessions. The dashed line in ***C*** represents the significance level at *p* = 0.05. Error bars indicate standard error.

With the selected values of *w_1_*, *w_2_*, and γ, we performed 20 simulations for each subject (for each of the *x* and *y* dimensions) to produce the dart-landing and self-estimated positions during the bowling observation session. To start each simulation, the initial value of motor command in the first trial was determined as an average of the self-estimated positions exhibited by a subject in the nVF1 session of the experiment (see Eq. 2). The median of the 20 simulations were averaged over the subjects to plot the blue trace in Fig. 6*A*. Next, the same parameter set was used to simulate the darts observation session.

#### Estimation of deterioration in nVF behavior during the darts observation session

The darts landing position deviated from the center in the darts observation session, whereas the self-estimated position remained close to the center (see Fig. 4). To model this behavior, we included the deterioration factors β_CON_ to the controller and β_FOR_ to the outcome forward model in Eqs. 1 and 2, respectively. Each factor was assumed to be either additive or multiplicative (see Discussion for details). Thus, we considered four possible deterioration models, *C^×^F^×^*, *C^×^F^+^*, *C^+^F^×^*, and *C^+^F^+^*, in which *C* and *F* represent controller and outcome forward model, respectively, and + and × represent additive and multiplicative deteriorations. The four models are described as follows:

*C^×^F^×^* model (Extended Data 3) considers a multiplicative deterioration to both the controller ($\beta_{CON}^{k}=\left[\begin{array}{cc} \beta_{CON}^{k\_x} & 0\\ 0 & \beta_{CON}^{k\_y} \end{array}\right]$) and the outcome forward model ($\beta_{FOR}^{k}=\left[\begin{array}{cc} \beta_{FOR}^{k\_x} & 0\\ 0 & \beta_{FOR}^{k\_y} \end{array}\right]$) such that
  (4) $$M(i)=(I-\beta_{CON}^{k})\cdot M(i-1)+\gamma \cdot D_{se}(i-1)+\delta_{M}\left[\begin{array}{c} \varepsilon^{x}\\ \varepsilon^{y} \end{array}\right]$$
  (5) $$D_{se}(i)=(I-\beta_{FOR}^{k})\cdot M(i)+\delta_{SE}[\begin{array}{c} \eta^{x}\\ \eta^{y} \end{array}].$$
*C^+^F^×^* model (Extended Data 4) considers an additive deterioration to the controller ($\beta_{CON}^{k}=\left[\begin{array}{c} \beta_{CON}^{k\_x}\\ \beta_{CON}^{k\_y} \end{array}\right]$) and a multiplicative deterioration to the outcome forward model ($\beta_{FOR}^{k}=\left[\begin{array}{cc} \beta_{FOR}^{k\_x} & 0\\ 0 & \beta_{FOR}^{k\_y} \end{array}\right]$) such that 
  (6) $$M(i)=M(i-1)+\gamma \cdot D_{se}(i-1)+\beta_{CON}^{k}+\delta_{M}\left[\begin{array}{c} \varepsilon^{x}\\ \varepsilon^{y} \end{array}\right],$$
  (7) $$D_{se}(i)=(I-\beta_{FOR}^{k})\cdot M(i)+\delta_{SE}\left[\begin{array}{c} \eta^{x}\\ \eta^{y} \end{array}\right].$$
*C^×^F^+^* model (Extended Data 5) considers a multiplicative deterioration to the controller ($\beta_{CON}^{k}=\left[\begin{array}{cc} \beta_{CON}^{k\_x} & 0\\ 0 & \beta_{CON}^{k\_y} \end{array}\right]$) and an additive deterioration to the outcome forward model ($\beta_{FOR}^{k}=\left[\begin{array}{c} \beta_{FOR}^{k\_x}\\ \beta_{FOR}^{k\_y} \end{array}\right]$) such that
  (8) $$M(i)=(I-\beta_{CON}^{k})\cdot M(i-1)+\gamma \cdot D_{se}(i-1)+\delta_{M}\left[\begin{array}{c} \varepsilon^{x}\\ \varepsilon^{y} \end{array}\right],$$
  (9) $$D_{se}(i)=M(i)+\beta_{FOR}^{k}+\delta_{SE}\left[\begin{array}{c} \eta^{x}\\ \eta^{y} \end{array}\right].$$
*C^+^F^+^* model (Extended Data 6) considers an additive deterioration in both the controller ($\beta_{CON}^{k}=\left[\begin{array}{c} \beta_{CON}^{k\_x}\\ \beta_{CON}^{k\_y} \end{array}\right]$) and the outcome forward model ($\beta_{FOR}^{k}=\left[\begin{array}{c} \beta_{FOR}^{k\_x}\\ \beta_{FOR}^{k\_y} \end{array}\right]$) such that
  (10) $$M(i)=M(i-1)+\gamma \cdot D_{se}(i-1)+\beta_{CON}^{k}+\delta_{M}\left[\begin{array}{c} \varepsilon^{x}\\ \varepsilon^{y} \end{array}\right],$$
  (11) $$D_{se}(i)=M(i)+\beta_{FOR}^{k}+\delta_{SE}\left[\begin{array}{c} \eta^{x}\\ \eta^{y} \end{array}\right].$$

Eq. 3 was used in all four models to represent the motor execution process. $\beta_{CON}^{k}$ and $\beta_{FOR}^{k}$ (*k* = [1:5]) represent the deterioration values in the *k* th nVF block. We assumed that $\beta_{CON}^{k}$ and $\beta_{FOR}^{k}$ changed linearly from 0 during nVF1. Therefore, the values of $\beta_{CON}^{k}$ and $\beta_{FOR}^{k}$ were determined as $\beta_{CON}^{k}=\frac{(k-1)\cdot \beta_{CON}^{5}}{4}$ and $\beta_{FOR}^{k}=\frac{(k-1)\cdot \beta_{FOR}^{5}}{4}$, respectively. We thus estimated the set of free parameters, $\beta_{CON}^{5}$ and $\beta_{FOR}^{5}$, for each of the *x* and *y* dimensions, to best fit the change from nVF1 to nVF5 in both dart-landing and self-estimated positions for each model of each subject (see below for details).

The multiplicative deterioration parameters were varied in the range of [0.01,1] (zero representing the default system) at intervals of 0.01. The additive deterioration parameters were varied in the range of [–10,10] at intervals of 0.01. The Gaussian random values of *ε*, *η*, and *ξ* were drawn from a normal distribution with mean 0 and variance 1.

Next, in simulations with each of the four models, we used all the combinations of the two parameters, $\beta_{CON}^{5}$ and $\beta_{FOR}^{5}$, to see which explains the subject behavior best. As with the bowling session analysis, with each parameter set, we first performed 20 simulations to produce the dart-landing and self-estimated positions for each of the *x* and *y* dimensions. To start each simulation, the initial value of the motor command in the first trial was determined from the subject’s behavior in the experiment, as the average of his self-estimated positions in nVF1. We determined the $\beta_{CON}^{5}$ and $\beta_{FOR}^{5}$ for each model and for each subject, with which the mean residual (across 20 simulations) between the simulation and experimental data were minimum. The residual for each of the *x* and *y* dimensions was calculated as $\sqrt{\frac{{\left(\Delta D_{a}-\Delta {D^{\prime }}_{a}\right)}^{2}}{2}+\frac{{\left(\Delta D_{se}-\Delta {D^{\prime }}_{se}\right)}^{2}}{2}}$, where Δ*D_a_* and Δ*D_se_* are changes in the average dart-landing and self-estimated positions, respectively, of experimental data from nVF1 to nVF5. $\Delta {D^{\prime }}_{a}$ and $\Delta {D^{\prime }}_{se}$ represent the changes in the average dart-landing and self-estimated positions, respectively, of simulation data from nVF1 to nVF5. We chose this residual for model fitting, because, as described in the Introduction, we considered that changes in the dart-landing and self-estimated positions during the darts observation session would best reflect the effects of outcome prediction of observed actions on the experts’ motor system.

We compared the four models in terms of model-fitting accuracy and its ability to explain three key observations in our results: (1) the dart-landing position progressively deviates from the dartboard center; (2) the self-estimated position, however, remains bounded around the center; and (3) the self-estimated position shows a substantial correlation to the dart-landing position (see Results). The model-fitting accuracy was defined as the averaged residual over *x* and *y* dimensions.

### Code availability

The computer codes that were used to generate the results central to the conclusions of this study are available as extended data (Extended Data 1–8).

Legends for Extended Data **Extended Data 1. DataSimulation.m**. This program selects one of the seven simulation models (model 1–7) used in this study and plots the dart-landing and self-estimated position data simulated by the selected model.
**Extended Data 2. model1.m**. This program is an implementation of the simulation algorithm of the model to reproduce the bowling observation session data.
**Extended Data 3. model2.m**. This program is an implementation of the simulation algorithm of the *C^×^F^×^* model, which cannot reproduce the darts observation session data.
**Extended Data 4. model3.m**. This program is an implementation of the simulation algorithm of the *C^+^F^×^* model, which cannot reproduce the darts observation session data.
**Extended Data 5. model4.m**. This program is an implementation of the simulation algorithm of the *C^×^F^+^* model, which can reproduce the darts observation session data.
**Extended Data 6. model5.m**. This program is an implementation of the simulation algorithm of the *C^×^F^×^* model, which cannot reproduce the darts observation session data.
**Extended Data 7. model6.m**. This program is an implementation of the simulation algorithm of the *C^×^* model, which cannot reproduce the darts observation session data.
**Extended Data 8. model7.m**. This program is an implementation of the simulation algorithm of the *F^+^* model, which can reproduce the darts observation session data. Download Extended Data 1, ZIP file

## Results

### Subject behavior: outcome prediction improvement deteriorates self-estimation

In the beginning of the trainable observation sessions of Experiment 1 (before the first OP block), the experts’ darts performance and self-estimation were comparable between the darts observation and bowling observation sessions. The subject-averaged darts error (Fig. 2) in VF1 was 2.11 ± 0.79 cm (mean ± SD) and 2.58 ± 1.1 cm in the darts and bowling observation sessions, respectively. On the other hand, the subject-averaged self-estimation error (Fig. 2) in nVF1 was 2.59 ± 0.79 and 2.97 ± 0.67 cm in the darts observation and bowling observation sessions, respectively. There were no significant differences in the darts error in VF1 between the darts and bowling observation sessions (*t*(15) = –1.635, *p* = 0.123)^a^ or the self-estimation error in nVF1 (*t*(15) = –1.846, *p* = 0.085).^b^

Fig. 3 shows the changes in the outcome prediction of observed darts and bowling actions during the OP task (left yellow panel) and changes in the self-estimation error and darts error from motor action task (right cyan panel) through the trainable observation sessions (Experiment 1). We separately performed a one-sample *t* test to examine the changes in the outcome prediction, the self-estimation error, and the darts error during the darts observation and bowling observation sessions of Experiment 1. The significance level was set to *p* = 0.025 considering multiple comparison among these two observation sessions for each *t* test. The experts showed significant increases in their ability to predict the outcomes of the novices’ actions in both darts (left red bar, *t*(15) = 6.544, *p* = 9.245 × 10^−6^)^c^ and bowling (left blue bar, *t*(15) = 8.251, *p* = 5.874 × 10^−6^).^d^ This improvement in outcome prediction was accompanied by a significant increase in the experts’ darts error during the darts observation session (right red bar, *t*(15) = 5.096, *p* = 1.315 × 10^−4^),^e^ but not during the bowling observation session (right blue bar, *t*(15) = –0.110, *p* = 0.914),^f^ as previously reported by us (Ikegami and Ganesh, 2014). Crucially for this study, a significant increase in the experts’ self-estimation error was observed only during the darts observation session (middle red bar, *t*(15) = 2.614, *p* = 0.020),^g^ but not during the bowling observation session (right blue bar, *t*(15) = –0.198, *p* = 0.845).^h^ The absence of an increase in self-estimation error and darts error during the bowling observation sessions shows that the self-estimation error increase during the darts observation sessions is not due to time drifts, fatigue, or attention loss.

Next, through the untrainable observation session (Experiment 2), we could conclusively show that the increases in the self-estimation error and darts error in Experiment 1 were due to changes in the outcome prediction ability of the experts and not simply the observation of the novices’ darts actions. In Experiment 2, the experts were again asked to watch and predict novices’ dart throws in the OP task but without prior information about the action goal (aiming for the board center) and without the trial-by-trial oral outcome feedbacks. These modifications were expected to attenuate the prediction errors available to the experts and thus suppress the improvement in their prediction ability (see Methods; Ikegami and Ganesh, 2014).

In the absence of these prediction errors, the outcome prediction improvement in the untrainable darts observation session of Experiment 2 (left gray bar in Fig. 3) was significantly lower than in the trainable darts observation session of Experiment 1 (two-sample *t* test, *t*(29) = 3.820, *p* = 6.503 × 10^−4^).^i^ Crucially, with the attenuation in the outcome prediction improvement, there was no significant change in the experts’ self-estimation error (middle gray bar, *t*(15) = 0.920, *p* = 0.372)^j^ or darts error (right gray bar, *t*(15) = 0.107, *p* = 0.917)^k^ in Experiment 2, although across the subject population, the experts watched the same darts videos in the darts observation session as in Experiment 1. Therefore, Experiments 1 and 2 together suggest a causal relationship between outcome prediction of observed actions and self-estimation of one’s own actions. That is, when we made an intervention in an expert’s ability to predict outcomes of observed throws (allowed the experts to learn outcome prediction by providing relevant prediction feedbacks), the ability to estimate the outcome of his own throws was affected.

### Quantifying changes in the dart-landing and self-estimated positions

To check whether the deteriorations in the darts performance and self-estimation in the trainable darts observation session of Experiment 1 were due to changes in variance or position bias of the dart-landing and self-estimated positions (Fig. 2; see Methods), we examined whether these two behavioral metrics changed across nVF blocks and across observation sessions.

Repeated two-way ANOVAs (5 nVF blocks × 2 observation sessions) on the 2D standard deviations (see Methods) showed that neither the dart-landing position nor the self-estimated position significantly changed across blocks (*F*(4,60) = 0.908, *p* = 0.465; *F*(4,60) = 0.714, *p* = 0.585)^l,m^ or observation sessions (*F*(1,15) = 0.007, *p* = 0.932; *F*(1,15) = 0.523, *p* = 0.481).^l,m^ That is, the deterioration in the darts performance and self-estimation was not due to an increase in variance in the experts’ motor performance.

A repeated two-way ANOVA (5 nVF blocks × 2 observation sessions) on the self-estimated position bias exhibited no significant main effects of blocks (*F*(4,60) = 1.466, *p* = 0.224)^n^ and sessions (*F*(1,15) = 1.536, *p* = 0.234)^n^ (Fig. 4*A*). On the other hand, a repeated two-way ANOVA on the dart-landing position bias exhibited a substantial interaction effect between the blocks and sessions that tended to significance (*F*(4,60) = 2.247, *p* = 0.075)^o^ (Fig. 4*B*). We thus separately performed a one-way ANOVA for each observation session to examine the changes over the nVF blocks. There was a significant main effect in the darts observation session (*F*(4,60) = 2.926, *p* = 0.028)^p^ but not in the bowling observation session (*F*(4,60) = 1.807, *p* = 0.139).^q^ *Post hoc* Tukey’s tests showed that the dart-landing position bias in the darts observation session was significantly larger in nVF3 (*p* = 0.035)^r^ than in nVF1 (red data, Fig. 4*B*).

To summarize, the above results suggested that the deterioration in the darts performance was largely driven by an increase in the dart-landing position bias. On the other hand, the self-estimated position remained bounded around the board center, consequently resulting in a progressive increase of the self-estimation error (difference between the dart-landing position and self-estimated position).

### Correlation between the dart-landing and self-estimated positions

The fact that the self-estimated position remains near the board center (magenta data, Fig. 4*B*) suggests the possibility that the darts experts randomly self-estimate a location near the board center. To investigate this possibility, we performed a Pearson’s correlation analysis between the dart-landing and self-estimated positions by each subject (see Methods) in the trainable observation sessions (Experiment 1). If the experts randomly estimate and do not use some aspect of their motor action to make the self-estimation, we expected an absence of correlation between the two. Fig. 4*D* shows data from a representative expert subject in the darts observation session. Fig. 4*C* shows the subject-averaged correlation coefficients averaged across the *x* and *y* dimensions in the bowling and darts observation sessions. We found a strong correlation between the dart-landing and self-estimation positions in the darts observation (*r* = 0.645 ± 0.152, *t*(15) = 9.338, *p* = 1.222 × 10^−7^)^s^ and the bowling observation sessions (*r* = 0.541 ± 0.214, *t*(15) = 4.734, *p* = 2.662 × 10^−4^)^t^, both of which were significantly larger than *r* = 0.279 (dotted line: significance level at *p* = 0.05). This result suggested that the experts used some aspect of their motor action to make the self-estimation and did not just randomly estimate a location near the board center.

### Key observations from experiments

Taken together, our behavioral experiments thus revealed three key observations defining how the self-estimation and the darts performance by the darts experts were affected by an improvement in the outcome prediction of novice dart throws: (1) Increase in dart-landing position bias: the dart-landing position progressively deviates from the board center; (2) constant self-estimated position bias: the self-estimated position remains bounded around the board center; and (3) correlation between dart-landing and self-estimated positions: the self-estimated position shows a substantial correlation with the dart-landing position.

Next, to show that the same forward model is involved in both self-estimation (of one’s own actions) and outcome prediction (of other’s actions), we show that the deterioration in the self-estimation and the darts performance is due to the change in the experts’ forward model. We used a model-based analysis for this demonstration.

### Modeling the expert darts performance

Eqs. 1, 2, and 3 represent our simplified model of the subjects’ behavior in our experiment. This model (Fig. 5; see Methods for details) assumes that for an intended darts goal, a subject plans the required motor command **M** in the *i* th trial using a controller (Eq. 1). While the motor command is sent to the musculoskeletal system that determines the dart-landing position **D_a_** in the *i* th trial (Eq. 3), the same motor command is also sent to the outcome forward model that provides the self-estimated position **D_se_** in the *i* th trial (Eq. 2). This idea, that a common motor command (or its efferent copy) is used for both action performance and self-estimation by humans, is popular in motor control literature (Haruno et al., 2001; Todorov and Jordan, 2002; Shadmehr and Wise, 2005; Franklin and Wolpert, 2011; Scott, 2012) and agrees with our experimental data (specifically key observation 3).

### Explaining the darts performance and self-estimation in the bowling observation session

First, the bowling observation session data of Experiment 1 was simulated for each subject using Eqs. 1, 2, and 3 and with γ = −0.5, *w_1_* =0.5, and *w_2_* = 1 (see Methods for details on parameter choice and Results for a parameter sensitivity analysis). The across-subjects average of the simulations is shown in Fig. 6*A*. Two-way ANOVA (5 nVF blocks × 2 position metrics: dart-landing and self-estimated positions) of the simulated data showed that neither the simulated dart-landing nor self-estimated position biases changed across the nVF blocks, and they remained near the board center (Fig. 6*A*; no main effects of blocks, *F*(4,60) = 0.286, *p* = 0.886, and no interaction effects, *F*(4,60) = 0.346, *p* = 0.846).^u^ In addition, a strong correlation between the self-estimated position and the dart-landing position (Fig. 6*C*; *r* = 0.61 ± 0.02 (mean ± SD), *t*(15) = 71.411 *p* = 2.051 × 10^−20^)^v^ was successfully reproduced. The state–space model could thus reproduce the main aspects of the experimental results in the bowling observation session (Extended Data 2).

### Explaining the darts performance and self-estimation in the darts observation session

To explain the changes in the dart-landing and self-estimated positions in the trainable darts observation session (Fig. 4*B*) of Experiment 1, we considered four possible deterioration models of the subject’s motor system, namely *C^×^F^×^*, *C^+^F^×^*, *C^×^F^+^*, and *C^×^F^×^* (see Methods for the mathematical definitions). Each model assumes either a multiplicative (represented by ×) or additive (+) deterioration in the controller (*C*) represented by Eq. 1 or outcome forward model (*F*) represented by Eq. 2. For each model, we simulated the darts performance and self-estimation for every subject to select two parameters, $\beta_{CON}^{5}$ (deterioration factor to the controller) and $\beta_{FOR}^{5}$ (deterioration factor to the outcome forward model), that give the best model-fitting accuracy (see Methods).

A one-way ANOVA on the model-fitting accuracy showed a significant main effect (*F*(3,45) = 12.97, *p* = 3.090 × 10^−6^)^w^ of the model type. *Post hoc* Tukey’s tests revealed that the *C^×^F^×^* model [0.93 ± 0.31 (mean ± SD) cm] was substantially worse than the *C^+^F^×^* (0.78 ± 0.23 cm, *p* = 0.051),^x^ *C^×^F^+^* (0.68 ± 0.19 cm, *p* = 0.001),^y^ and *C^+^F^+^* (0.59 ±0.15 cm, *p* = 0.001)^z^ models. On the other hand, there was no clear difference between the remaining three models (*C^+^F^×^* vs. *C^×^F^+^* models: *p* = 0.327; *C^×^F^+^* vs. *C^+^F^+^* models: *p* = 0.402).^aa,bb^ We thus rejected only the *C^×^F^×^* model in terms of model-fitting accuracy (Extended Data 4).

Next, to examine which of the remaining three models explains our data best, we checked whether the models can explain the bias in the darts landing position (key observation 1) and lack of bias in the self-estimated position (key observation 2) in our experimental data. We performed two-way ANOVAs (5 nVF blocks × 2 position metrics) for this. A significant interaction effect was observed across the factors in all the three models (*C^+^F^×^*: *F*(4,60) = 6.243, *p* = 2.872 × 10^−4^; *C^×^F^+^*: *F*(4,60) = 5.366, *p* = 9.229 × 10^−4^; *C^+^F^+^*: *F*(4,60) = 2.694, *p* = 0.039).^cc,dd,ee^ We thus checked simple effect of blocks for each of the dart-landing and self-estimated positions.

The *C^+^F^×^* model and the *C^+^F^+^* model showed significant simple main effects of blocks (F(4,60) ≥ 11.320, *p* ≤ 6.565 × 10^−7^^cc,ee^, see Table 1) for both the dart-landing position and the self-estimated position. *Post hoc* Tukey’s tests revealed that both the dart-landing position (nVF1 vs. nVF5, *p* ≤ 9.519 × 10^−4^)^ff^ and the self-estimated position biases (nVF1 vs. nVF5, *p* ≤ 9.073 × 10^−4^)^gg^ significantly increased in these two models. The increase in the self-estimated position bias violates key observation 2, indicating that both the *C^+^F^×^* (Extended Data 4) and *C^+^F^+^* (Extended Data 6) models are not suitable to explain our data. On the other hand, the *C^×^F^+^* model satisfied key observations 1 and 2. The significant simple main effects of blocks was found for the dart-landing position bias (*F*(4,60) = 4.258 × 10^−5^, *p* < 10^−4^),^dd^ but not for the self-estimated position bias (*F*(4,60) = 0.212, *p* = 0.931).^dd^ The bias in the dart-landing position significantly increased (hence darts performance deteriorated) between the nVF1 and nVF5 blocks (*p* = 9.519 × 10^−4^, Fig. 6*B*),^ff^ while the self-estimated position bias did not change.

**Table 1.** Summary of statistical analysis

Location	Dependent variable	Type of test	Statistic	Confidence
a	Darts errors in the VF1 during the darts and bowling observation sessions in Exp. 1	Paired *t* test	*t*(15) = –1.635	*p* = 0.123, CI = –1.08/0.14
b	Self-estimation errors in the VF1 during the darts and bowling observation sessions in Exp. 1	Paired *t* test	*t*(15) = –1.846	*p* = 0.085, CI = –0.822/0.059
c	Change in the outcome prediction of the observed darts actions in Exp. 1	One-sample *t* test	*t*(15) = 6.544	*p* = 9.245 × 10^−6^ (corrected *p* = 0.025), CI = 7.242/16.138
d	Change in the outcome prediction of the observed bowling actions in Exp. 1	One-sample *t* test	*t*(15) = 8.251	*p* = 5.874 × 10^−6^ (corrected *p* = 0.025), CI = 5.981/11.150
e	Change in the darts error during the darts observation session in Exp. 1	One-sample *t* test	*t*(15) = 5.096	*p* = 1.315 × 10^−4^ (corrected *p* = 0.025), CI = 0.51/1.504
f	Change in the darts error during the bowling observation session in Exp. 1	One-sample *t* test	*t*(15) = –0.110	*p* = 0.914 (corrected *p* = 0.025), CI = –0.789/0.722
g	Change in the self-estimated error during the darts observation session in Exp. 1	One-sample *t* test	*t*(15) = 2.614	*p* = 0.020 (corrected *p* = 0.025), CI = 0.033/1.370
h	Change in the self-estimated error during the bowling observation session in Exp. 1	One-sample *t* test	*t*(15) = –0.198	*p* = 0.845 (corrected *p* = 0.025), CI = –0.710/0.605
i	Changes in the outcome prediction of the observed darts actions in Exps. 1 and 2	Two-sample *t* test	*t*(29) = 3.820	*p* = 6.503 × 10^−4^, CI = 4.054/13.397
j	Change in the self-estimated error in Exp. 2	One-sample *t* test	*t*(15) = 0.920	*p* = 0.372, CI = –0.231/0.580
k	Change in the darts error in Exp. 2	One-sample *t* test	*t*(15) = 0.107	*p* = 0.917, CI = –0.673/0.744
l	2D standard deviations of the dart-landing position across nVF blocks and observation sessions in Exp. 1	Repeated two-way ANOVA	Block_F(4,60) = 0.908, Session_F(1,15) = 0.007, Interaction_F(4,60) = 0.822	*p* = 0.465, Block_η_p_^2^ = 0.057; *p* = 0.932, Session_η_p_^2^ = 4.976 × 10^−4^; *p* = 0.517, Interaction_η_p_^2^ = 0.052
m	2D standard deviations of the self-estimated position across nVF blocks and observation sessions in Exp. 1	Repeated two-way ANOVA	Block_F(4,60) = 0.714, Session_F(1,15) = 0.523, Interaction_F(4,60) = 1.959	*p* = 0.585, Block_η_p_^2^ = 0.046; *p* = 0.481, Session_η_p_^2^ = 0.034; *p* = 0.112, Interaction_η_p_^2^ = 0.116
*n*	Self-estimated position biases across nVF blocks and observation sessions in Exp. 1	Repeated two-way ANOVA	Block_F(4,60) = 1.466, Session_F(1,15) = 1.536, Interaction_F(4,60) = 0.483	*p* = 0.224, Block_η_p_^2^ = 0.089; *p* = 0.234, Session_η_p_^2^ = 1.024 × 10^−8^; *p* = 0.748, Interaction_η_p_^2^ = 0.031
o	Dart-landing position biases across nVF blocks and observation sessions in Exp. 1	Repeated two-way ANOVA	Block_F(4,60) = 2.693 Session_F(1,15) = 0.351 Interaction_F(4,60) = 2.247	*p* = 0.039, Block_η_p_^2^ = 0.1552; *p* = 0.562, Session_η_p_^2^ = 0.023; *p* = 0.075, Interaction_η_p_^2^ = 0.130
*p*	Dart-landing position biases across nVF blocks during the darts observation session in Exp. 1	Repeated one-way ANOVA	F(4,60) = 2.926	*p* = 0.028, η_p_^2^ = 0.163
q	Dart-landing position biases across nVF blocks during the bowling observation session in Exp. 1	Repeated one-way ANOVA	F(4,60) = 1.807	*p* = 0.139, η_p_^2^ = 0.108
r	Dart-landing position biases in nVF1 and nVF3 during the darts observation session in Exp. 1	*Post hoc* Tukey’s test	q_(k = 5, df = 60)_ = 4.191	*p* = 0.035
s	Correlation coefficients between the dart-landing and self-estimation positions during the darts observation session in Exp. 1	One-sample *t* test	*t*(15) = 9.338	*p* = 1.222 × 10^−7^, CI = 0.282/0.450
*t*	Correlation coefficients between the dart-landing and self-estimation positions during the bowling observation session in Exp. 1	One-sample *t* test	*t*(15) = 4.734	P = 2.662 × 10^−4^, CI = 0.144/0.379
u	Simulated position biases across nVF blocks and position metrics during the bowling observation session	Repeated two-way ANOVA	Block_F(4,60) = 0.286, Position_F(1,15) = 107.702, Interaction_F(4,60) = 0.346	*p* = 0.886, Block_η_p_^2^ = 0.0187; *p* = 3.060 × 10^−8^, Position_η_p_^2^ = 0.878; *p* = 0.846, Interaction_η_p_^2^ = 0.023
v	Correlation coefficients between the simulated dart-landing and self-estimation positions during the bowling observation session	One-sample *t* test	*t*(15) = 71.411	*p* = 2.051 × 10^−20^, CI = 0.319/0.339
w	Model-fitting accuracies across the deterioration models	Repeated one-way ANOVA	F(3,45) = 12.972	*p* = 3.090 × 10^−6^, η_p_^2^ = 0464
x	Model-fitting accuracies in *C^×^F^×^* and *C^+^F^×^* models	*Post hoc* Tukey’s test	q_(k = 4, df = 45)_ = 3.768	*p* = 0.0511
y	Model-fitting accuracies in *C^×^F^×^* and *C^×^F^+^* models	*Post hoc* Tukey’s test	q_(k = 4, df = 45)_ = 6.197	*p* = 0.001
z	Model-fitting accuracies in *C^×^F^×^* and *C^+^F^+^* models	*Post hoc* Tukey’s test	q_(k = 4, df = 45)_ = 8.428	*p* = 0.001
aa	Model-fitting accuracies in *C^+^F^×^* and *C^×^F^+^* models	*Post hoc* Tukey’s test	q_(k = 3, df = 45)_ = 2.429	*p* = 0.327
bb	Model-fitting accuracies in *C^×^F^+^* and *C^+^F^+^* models	*Post hoc* Tukey’s test	q_(k = 4, df = 45)_ = 2.231	*p* = 0.402

cc	Simulated position biases across nVF blocks and position metrics for *C^+^F^×^* model	Repeated two-way ANOVA	Block_F(4,60) = 25.845, Position_F(1,15) = 29.022, Interaction_F(4,60) = 6.243, Simple main effect of blocks: Dart-landing_F(4,60) = 21.436, Self-estimated_F(4,60) = 17.819	*p* = 7.549 × 10^−5^, Block_η_p_^2^ = 0.633; *p* = 1.775 × 10^−12^, Position_η_p_^2^ = 0.659; *p* = 2.872 × 10^−4^, Interaction_η_p_^2^ = 0.294; *p* = 5.099 × 10^−11^, Dart-landing_η_p_^2^ = 0.588; *p* = 1.088 × 10^−9^, Self-estimated_η_p_^2^ = 0.543
dd	Simulated position biases across nVF blocks and position metrics for *C^×^F^+^* model	Repeated two-way ANOVA	Block_F(4,60) = 8.033, Position_F(1,15) = 16.645, Interaction_F(4,60) = 5.366, Simple main effect of blocks: Dart-landing_F(4,60) = 7.739, Self-estimated_F(4,60) = 0.212	*p* = 2.962 × 10^−5^, Block_η_p_^2^ = 0.349; *p* = 9.858 × 10^−4^, Position_η_p_^2^ = 0.526; *p* = 9.229 × 10^−4^, Interaction_η_p_^2^ = 0.2635; *p* = 4.258 × 10^−5^, Dart-landing_η_p_^2^ = 0.340; *p* = 0.931, Self-estimated_η_p_^2^ = 0.014
ee	Simulated position biases across nVF blocks and position metrics for *C^+^F^+^* model	Repeated two-way ANOVA	Block_F(4,60) = 26.752, Position_F(1,15) = 9.673, Interaction_F(4,60) = 2.694, Simple main effect of blocks: Dart-landing_F(4,60) = 16.260, Self-estimated_F(4,60) = 11.320	*p* = 9.295 × 10^−13^, Block_η_p_^2^ = 0.641; *p* = 0.007, Position_η_p_^2^ = 0.392; *p* = 0.039, Interaction_η_p_^2^ = 0.152; *p* = 4.501 × 10^−9^, Dart-landing_η_p_^2^ = 0.520; *p* = 6.565 × 10^−7^, Self-estimated_η_p_^2^ = 0.430
ff	Simulated dart-landing position biases in nVF1 and nVF5 for each of *C^+^F^×^*, *C^×^F^+^*, and *C^+^F^+^* model	*Post hoc* Tukey’s test	**C*^*+*^*F*^*×*^*_**q_(k = 5, df = 60)_ = 11.230, **C*^*×*^*F*^*+*^*_**q_(k = 5, df = 60)_ = 6.957, **C*^*+*^*F*^*+*^*_**q_(k = 5, df = 60)_ = 9.660	*p* = 9.073 × 10^−4^, *p* = 9.519 × 10^−4^, *p* = 9.073 × 10^−4^
gg	Simulated self-estimated position biases in nVF1 and nVF5 for each of *C^+^F^×^* and *C^+^F^+^* model	*Post hoc* Tukey’s test	**C*^*+*^*F*^*×*^*_**q_(k = 5, df = 60)_ = 10.719, **C*^*+*^*F*^*+*^*_**q_(k = 5, df = 60)_ = 8.527	*p* = 9.073 × 10^−4^, *p* = 9.076 × 10^−4^
hh	Correlation coefficients between the simulated dart-landing and self-estimation positions for *C^×^F^+^* model	Paired *t* test	*t*(15) = 68.514	*p* = 3.810 × 10^−20^, CI = 0.301/0.320
ii	Simulated position biases across nVF blocks and position metrics for **F*^*+*^* model	Repeated two-way ANOVA	Block_F(4,60) = 5.825, Position_F(1,15) = 16.536, Interaction_F(4,60) = 7.733, Simple main effect of blocks: Dart-landing_F(4,60) = 7.456, Self-estimated_F(4,60) = 0.835	*p* = 4.988 × 10^−4^, Block_η_p_^2^ = 0.280; *p* = 0.001, Position_η_p_^2^ = 0.524; *p* = 4.290 × 10^−5^, Interaction_η_p_^2^ = 0.340; *p* = 7.558 × 10^−9^, Dart-landing_η_p_^2^ = 0.332; *p* = 0.508, Self-estimated_η_p_^2^ = 0.05
jj	Correlation coefficients between the simulated dart-landing and self-estimation positions for **F*^*+*^* model	Paired *t* test	*t*(15) = 73.051	*p* = 1.460 × 10^−20^, CI = 0.314/0.333
kk	Correlation coefficients between the simulated dart-landing and self-estimation positions for *C^×^* model	Paired *t* test	*t*(15) = 35.622	*p* = 6.539 × 10^−16^, CI = 0.303/0.342
ll	Simulated position biases across nVF blocks and position metrics for *C^×^* model	Repeated two-way ANOVA	Block_F(4,60) = 8.680, Position_F(1,15) = 15.107, Interaction_F(4,60) = 0.733,	*p* = 1.350 × 10^−5^, Block_η_p_^2^ = 0.367; *p* = 0.002, Position_η_p_^2^ = 0.502; *p* = 0.573, Interaction_η_p_^2^ = 0.047
mm	Model fitting accuracies of *C^×^F^+^* model with values of γ from 0 to –1	Repeated one-way ANOVA	F(10,150) = 26.078	η_p_^2^ = 0.6348, *p* = 10^−20^
nn	Model fitting accuracies of *C^×^F^+^* model with values of γ from 0 to –1	*Post hoc* Tukey’s test	For any pair of values obtained with γ ≤ –0.3, q_(k = 11, df = 150)_ ≤ 3.714	For any pair of values obtained with γ ≤ –0.3, *p* ≥ 0.235
oo	Self-estimated position biases of *C^×^F^+^* model with values of γ from 0 to –1	One-sample *t* test	For γ = 0, *t*(15) = –3.566, For γ ≤ –0.1, |*t*(15)| ≤ 1.829	For γ = 0, *p* = 0.003 (corrected *p* = 0.025); for γ ≤ –0.1, *p* ≥ 0.087 (corrected *p* = 0.025)
pp	Dart-landing position biases of *C^×^F^+^* model with values of γ from 0 to –1	One-sample *t* test	For γ ≤ –0.4, |*t*(15)| ≤ 2.698	for γ ≤ –0.4, *p* ≤ 0.017 (corrected *p* = 0.025)
qq	Correlation coefficients between the simulated dart-landing and self-estimation positions of *C^×^F^+^* model with values of γ from 0 to –1	One-sample *t* test	For all values of γ, *t*(15) ≥ 70.639	For all values of γ, *p* ≤ 2.413 × 10^−19^
rr	Values of $\beta_{CON}^{\;5}$ estimated by *C^×^F^+^* model with values of γ from 0 to –1	Repeated one-way ANOVA	F(10,150) = 6.358	η_p_^2^ = 0.298, *p* = 4.141 × 10^−8^
ss	Values of $\beta_{CON}^{\;5}$ estimated by *C^×^F^+^* model with values of γ from 0 to –1	*Post hoc* Tukey’s test	For any pair of values obtained with γ ≤ –0.3, q_(k = 11, df = 150)_ ≤ 2.714	For any pair of values obtained with γ ≤ –0.3, *p* ≥ 0.707
tt	Values of $\beta_{FOR}^{\;5}$ estimated by *C^×^F^+^* model with values of γ from 0 to –1	Repeated one-way ANOVA	F(10,150) = 2.432	η_p_^2^ = 0.1395, *p* = 0.010
uu	Values of $\beta_{FOR}^{\;5}$ estimated by *C^×^F^+^* model with values of γ from 0 to –1	*Post hoc* Tukey’s test	For any pair of values obtained with γ ≤ –0.1, q_(k = 11, df = 150)_ ≤ 2.998	For any pair of values obtained with γ ≤ –0.1, *p* ≥ 0.563
vv	Lag 1 autocorrelation coefficients of *x*- and *y*-axis data of self-estimated positions during the bowling observation session in Exp. 1	One-sample *t* test	*x*-axis_*t*(15) = –0.975, *y*-axis_*t*(15) = –1.784,	*p* = 0.345, CI = –0.088/0.033, *p* = 0.095, CI = –0.154/0.014
ww	Lag 1 autocorrelation coefficients of *x*- and *y*-axis data of self-estimated positions during the darts observation session in Exp. 1	One-sample *t* test	*x*-axis_*t*(15) = –0.429, *y*-axis_*t*(15) = –1.833	*p* = 0.674, CI = –0.099/0.066, *p* = 0.087, CI = –0.134/0.010

Finally, similar to the analysis of the experimental data, we calculated the correlation coefficient between the simulated dart-landing and the simulated self-estimated positions (Fig. 6*C*). We found that the *C^×^F^+^* model could reproduce strong correlations [*r* = 0.590 ± 0.018 (mean ± SD), *t*(15) = 68.514, *p* = 3.810 × 10^−20^]^hh^ across subjects, similar to our experimental data. The *C^×^F^+^* model thus also reproduced key observation 3.

Taken together, only the *C^×^F^+^* model could reproduce all three key observations in our experiment (Extended Data 5). These results suggest *C^×^F^+^* to be a good model of the deterioration of the subject’s motor system during the outcome prediction of observed actions in our darts observation sessions.

### Quantifying effects on the outcome forward model and the controller

The aim of our model-based analysis is to show that the forward model was indeed affected in our experiment. The fact that the *C^×^F^+^* model (which incorporates an additive deterioration of the forward model) explains our data supports this. However, it is still a concern whether the magnitude of deterioration in the forward model (namely $\beta_{FOR}^{5}$) is indeed significant. The estimated values of $\beta_{CON}^{5}$ in the *C^×^F^+^* model was 0.50 ± 0.30 (mean ± SD) and 0.34 ± 0.28 for *x* and *y* dimensions, respectively. On the other hand, the estimated values of $\beta_{FOR}^{5}$ were 1.78 ± 1.80 and 2.18 ± 2.05 for *x* and *y* dimensions, respectively. To show that these deteriorations estimated by the *C^×^F^+^* model are indeed significant with respect to explaining our experimental key observations, we evaluated how much either deterioration taken alone can explain the data.

For this, we resimulated the darts observation session data using the *C^×^F^+^* model twice, once with only the deterioration in the controller (the *C^×^* model), and once with only the deterioration in the outcome forward model (the *F^+^* model). The estimated values of $\beta_{CON}^{5}$ and $\beta_{FOR}^{5}$ in the *C^×^F^+^* model were used for the *C^×^* and *F^+^* models, respectively.

The *F^+^* model successfully reproduced all three key observations (Extended Data 8). The simulated dart-landing position bias significantly increased (simple main effect of blocks: *F*(4,60) = 7.456, *p* = 7.558 × 10^−9^),^ii^ while the simulated self-estimated position bias did not change (*F*(4,60) = 0.835, *p* = 0.508).^ii^ The two positions showed a significant trial-by-trial correlation (*r* = 0.602 ± 0.017, *t*(15) = 73.051, *p* = 1.460 × 10^−20^).^jj^ On the other hand, while the *C^×^* model, that did not include a deterioration in the forward model could reproduce the correlation between the dart-landing and self-estimated positions (*r* = 0.601 ± 0.035, *t*(15) = 35.622, *p* = 6.539 × 10^−20^^kk^, key observation III), it could not satisfy key observations 1 and 2 (Extended Data 7). The simulated dart-landing bias increased, while the self-estimated position bias decreased (main effect of blocks: *F*(4, 60) = 8.680, *p* = 1.350 × 10^−5^)^ll^ with no interaction effects (*F*(4,60) = 0.733, *p* = 0.573).^ll^ These results show that the deterioration in the outcome forward model ($\beta_{FOR}^{5}$) is critical to reproduce the experimental results.

### Sensitivity analysis of the *C^×^F^+^* model to change in learning rate

The learning rate, *γ*, determines how much the motor command in each throw is modified by the feedback (Eq. 8 in Methods). The simulation results presented here were performed with a predetermined learning rate of γ=−0.5 (see Methods). To ensure that this choice does not bias the simulations, we examined the sensitivity of the simulation results to the selection of the learning rate. For this, we simulated the *C^×^F^+^* model for every value of *γ* from 0 to –1 (in decrements of 0.1) and analyzed the model-fitting accuracy with each value.

**Figure 7.**
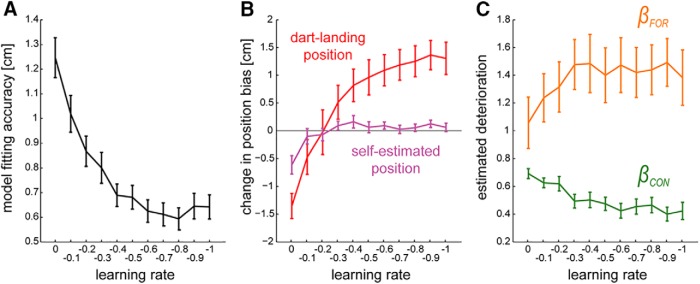
Sensitivity analysis of the *C^×^F^+^* model to change in learning rate. ***A***, The model-fitting accuracy obtained with each learning rate. ***B***, Simulation result of the changes in dart-landing (red) and self-estimated (magenta) position biases for each learning rate. ***C***, Estimated values of *β_CON_* (green) and *β_FOR_* (orange) for each learning rate. The values averaged over *x* and *y* dimensions were plotted. Error bars indicate standard error.

Fig. 7*A* shows the model-fitting accuracy obtained with each *γ*. The residuals drastically reduced as *γ* decreased from 0 to –0.2 (repeated one-way ANOVA: *F*(10, 150) = 26.078, *p* = 10^−20^)^mm^ and plateaued for γ≤−0.3 (*post hoc* Tukey’s test: *p* ≥ 0.235, see Table 1).^nn^

Fig. 7*B* shows the changes (from nVF1 to nVF5) in the dart-landing (red line) and self-estimated (magenta line) position biases with the change in *γ*. We performed *t* tests for the data simulated with each *γ* to examine whether the change in each of these two position biases is larger than zero (significance level was set to *p* = 0.025 considering multiple comparison among the two position biases). The self-estimated position bias did not show significant increase (*t* tests, *p* > 0.05, see Table 1)^oo^ for all values of *γ* except *γ* = 0, while the dart-landing position bias showed significant increase (*t* tests, *p* ≤ 0.017)^pp^ for γ≤−0.4. The strong correlation between the dart-landing and self-estimated positions (*r* > 0.55, *p* ≤ 2.413 × 10^−20^)^qq^ could be reproduced by all values of *γ*.

Fig. 7*C* shows the deterioration factors $\beta_{CON}^{5}$ and $\beta_{FOR}^{5}$ estimated with each *γ*. The shown values are averaged across the *x* and *y* dimensions. $\beta_{CON}^{5}$ decreased (*F*(10, 150) = 6.358, *p* = 4.141 × 10^−8^)^rr^ with a decrease in *γ*, but plateaued with γ≤−0.3 (*post hoc* Tukey’s test: *p* ≥ 0.707, see Table 1).^ss^ $\beta_{FOR}^{5}$ increased with an increase in *γ* (repeated one-way ANOVA: *F*(10, 150) = 2.432, *p* < 0.01)^tt^ but plateaued for γ≤−0.1 (*post hoc* Tukey’s test: *p* > 0.563).^uu^

Overall, we observed that for a value of γ≤−0.3, our simulation results using the *C^×^F^+^* model were robust to the selection of the learning parameter and could qualitatively reproduce all three key observations from the data.

In summary, the model-based analyses showed that an improvement in the outcome prediction by the darts experts affects their outcome forward model. Our experimental and simulation results together suggest that the forward model is involved in both the outcome estimation of observed actions and the self-estimation of one’s own actions.

## Discussion

Multiple studies over the last decades have proposed that humans use forward models to predict the sensory outcomes of not just self-generated movements (Wolpert et al., 1995; Desmurget and Grafton, 2000; Christensen et al., 2007; Miall et al., 2007) but also visually (Kilner et al., 2007; Friston et al., 2011; Kilner, 2011; Clark, 2013; Pickering and Clark, 2014) and haptically (Ganesh et al., 2014; Takagi et al., 2017) observed actions performed by others. However, experimental proof for showing that the forward models help us in predicting outcomes of self-generated as well as observed actions has remained absent (Pickering and Clark, 2014). In this study, using experiments and modeling of the darts experts’ behavior, we could show that, first, watching a novice dart thrower leads to deterioration in an expert’s ability to estimate his own dart throws. Second, and critically, the deterioration is present only when an expert’s ability to predict the outcome of the novice’s dart throws changes (improves; i.e., in the trainable darts observation session). Third, deterioration in the self-estimation can be explained only by a substantial change in the outcome forward model of the expert. This causal relation between outcome prediction of observed actions and one’s own outcome forward model supports the involvement of the same forward model in both the outcome prediction of observed actions and the outcome estimation of one’s own actions.

Similar to previous works (Thoroughman and Shadmehr, 2000; Donchin et al., 2003; van Beers, 2009; Yokoi et al., 2011), we used a simplified state–space model in the study that does not consider body dynamics but estimates the internal state of the motor system as a whole to specify a motor command corresponding to the dart-landing position on the dartboard. We used a minimalistic modeling approach to show that the popular forward model-controller structure (as in Fig. 5) is able to explain our results with sufficient detail. Our model is based on the following four assumptions.

First, we assumed that a common motor command is used for both action production and self-estimation. This assumption is based on the widely accepted view that our motor system utilizes an *efferent copy* of the motor command to estimate the sensory consequence and the future states of the system using a forward model (Kawato, 1999; Wolpert and Ghahramani, 2000; Shadmehr and Wise, 2005; Vaziri et al., 2006; Franklin and Wolpert, 2011). Furthermore, we observed a significant correlation between the self-estimated position and the dart-landing position (Fig. 4*A*, *C*), which supports this assumption.

Second, we assumed the noise characteristics to be the same between the bowling and darts observation sessions. The gradual deviation of the dart-landing positions over the nVF blocks (nonstationarity) impedes estimation of the noise characteristics of the system in the darts observation session. However, the variances of the dart-landing position and self-estimated position were found to be same in every nVF block (10 trials) between the darts and bowling observation sessions, suggesting similar noise characteristics.

Third, we assumed that the learning rate was not affected by the darts observation, and hence subjects used the same learning rate in the bowling and darts observation sessions. This assumption was based on our observation of zero lag 1 autocorrelation of self-estimated positions, both the bowling observation sessions (*x*-axis: −0.028 ± 0.110, *t*(15) = −0.975, *p* = 0.345; *y*-axis: −0.070 ± 0.152, *t*(15) = −1.78, *p* = 0.095)^vv^ and the trainable darts observation sessions of experiment 1 (*x*-axis: −0.017 ± 0.150, *t*(15) = −0.429, *p* = 0.674; *y*-axis: −0.062 ± 0.131, *t*(15) = −1.833, *p* = 0.087).^ww^ This result indicates that the learning rates in our experiment were near-optimally tuned to the total noise of the planning and estimation noises ($\delta_{M}\left[\begin{array}{c} \varepsilon^{x}\\ \varepsilon^{y} \end{array}\right]+\delta_{SE}\left[\begin{array}{c} \eta^{x}\\ \eta^{y} \end{array}\right]$), possibly to minimize the trial-by-trial endpoint variability around the target center (van Beers, 2009; van Beers et al., 2013). In light of the fact that the noise characteristics were observed to be similar in our bowling and darts observation sessions, the lag 1 autocorrelation result suggests that the learning rates were also similar between across the two observation sessions.

Fourth, previous motor studies have considered a multiplicative (such as signal-dependent noise; Harris and Wolpert, 1998; Jones et al., 2002) or additive (such as sensory uncertainty) noise/deterioration to the motor system to explain the variability of motor output (Todorov and Jordan, 2002; Izawa et al., 2008; Izawa and Shadmehr, 2008). In our modeling, the motor commands represent the locations on the dartboard a subject aims for, and as the size of the dart board is small relative to the distance of the thrower from the darts board, we assume that the difference in the motor signals associated with throws to be minimal. We therefore consider only additive noise ($\delta_{M}$ in the controller, $\delta_{SE}$ in the forward model, and $\delta_{E}$ in the musculoskeletal system). However, we consider both an additive or multiplicative deterioration in the forward model and controller to explain the changes in the experts’ darts performance and self-estimation after outcome prediction. The choice of the additive deterioration was motivated by automatic imitation studies (Brass et al., 2000; Heyes, 2011) that have shown that the actions of an observer may be biased by observations of others. On the other hand, the multiplicative deterioration (in fact a forgetting factor) was introduced, considering the possibility that observation of other’s movements may affect the retention and utilization of motor memory (or previously learned motor command).

The *C^×^F^+^* model explained our results the best, and suggests that the outcome forward model is affected by an additive deterioration. The trial-by-trial modification of the motor command using this deteriorated self-estimation deviates the dart-landing position away from the board center (Eq. 3, key observation 1). On the other hand, because the self-estimation, but not the actual dart-landing position, is used as feedback to the motor system (Eq. 8), the motor command in the next trial is updated to counteract the bias in the self-estimated position, resulting in the self-estimated position being kept bounded around the board center (key observation 2). Finally, the fact that the same motor command is used for both the darts execution as well as the self-estimation leads to a correlation between the two (key observation 3).

An effect of the outcome prediction of observed actions on the outcome forward model suggests the presence of common neural processes that contribute to both the outcome prediction of observed actions performed by others and the self-estimation of one’s own actions. This result has several important implications. Primarily, neural activations during both action generation and action observation are properties associated with the so-called mirror neurons (di Pellegrino et al., 1992; Rizzolatti and Craighero, 2004; Rizzolatti and Sinigaglia, 2010). Our result supports the possible function of mirror neurons in the forward modeling of actions (Miall, 2003; Kilner et al., 2007; Yarrow et al., 2009; Friston, 2011; Friston et al., 2011). Although physiologic evidence for this remains absent, it is interesting to note that the posterior parietal cortex, which has been considered a candidate neural substrate of the forward model (Desmurget et al., 1999; Mulliken et al., 2008), includes areas where mirror neurons have been observed, specifically in the anterior intraparietal area and area PFG of macaques (Rizzolatti and Sinigaglia, 2010; Kilner and Lemon, 2013). The investigation of mirror neurons or neural circuits in regard to forward modeling of actions will be an important direction for the fields of cognitive and social neuroscience.

Moreover, the effects of observed actions on the forward model may be crucial in regard to the learning of affordance (Gibson, 1979), which has been proposed to occur via the learning of mappings between an action and its effect on the environment or outcome (Montesano et al., 2008; Thill et al., 2013). Our results suggest possible mechanisms to explain how affordances may be implicitly learned or modified through the observation of interactions by others with the environment (Tomasello, 1996).

Finally, our results can provide insight into the possible interaction between action estimation and action production in the human motor system (Pickering and Clark, 2014). The traditional view from motor neuroscience believes sensory prediction to be the core role of the forward model (Miall and Wolpert, 1996) and that the forward model involves neural mechanisms distinct from those for action generation, which in turn is handled by the so called *inverse model* that generates motor commands given certain state feedbacks and predictions (Wolpert and Kawato, 1998; Kawato, 1999; Shadmehr and Wise, 2005; Pickering and Garrod, 2013). Our model in this article falls under this category. On the other hand, recent proposals dismiss the belief that the inverse model is indispensable for motor control. They hypothesize the possibility of a single forward model that contributes to both, action prediction and motor command generation in the brain (Kilner et al., 2007; Friston et al., 2011; Pickering and Clark, 2014). A critical prediction of this hypothesis is that any change in the action estimation ability should be accompanied by a change in the subject’s ability to produce appropriate motor commands for an intended action (motor plan; Pickering and Clark, 2014). However, this was apparently not so in our study, where the variance and zero lag 1 autocorrelation of the self-estimated position were observed to remain unchanged between the bowling and darts observation sessions, suggesting that the noise characteristics and the learning rate were similar between the two. In addition to the *C^×^* model analysis, these results suggest that, although the forward model was obviously affected in our study, the controller, which generates the motor plan, did not suffer a substantial change. Our results thus support the traditional view, that of distinct mechanisms for action prediction and motor command generation. However, further studies are needed to clearly show this distinction.

In conclusion, our study provides new insights on how our motor behavior is shaped by actions performed by others; prediction of observed actions can modify one’s forward model, in turn affecting one’s motor actions that are planned based on self-estimations by the same forward model. The forward model, acting as an interface between the self and others, may thus be the key modulator of our social motor skills.

## Synthesis

Reviewing Editor: Bradley Postle, University of Wisconsin Decisions are customarily a result of the Reviewing Editor and the peer reviewers coming together and discussing their recommendations until a consensus is reached. When revisions are invited, a fact-based synthesis statement explaining their decision and outlining what is needed to prepare a revision will be listed below. The following reviewer(s) agreed to reveal their identity: Erhan Oztop, Isaac Kurtzer

Both reviews are fairly positive. The reviewers had a chance to see each other's reviews, and no concerns were raised, so I'm passing the reviews along here.

Reviewer 1:

The paradigm employed nicely shows the shared use of a‘effect’ forward model for both execution and estimation of others’ actions. The use of the forward model is different from the one used in motor control literature (as also stated by the authors), so I chose to use the phrase‘effect forward model'. Similar effect prediction mechanisms are also suggested for affordance and skill learning in the context of object manipulation (e.g. google affordance and skill learning). It may be interesting to link to that literature, because the forward model here looks more like an “effect prediction” system. Then of course, how this affects one's own action generation is to be answered as expanded below.

Overall, it does not come naturally come out why such a forward model would be involved in motor execution, especially in over-practiced ballistic movements (such as dart throwing). The current use of the model does not fit the usual “delay compensation” role. Some discussion about this would strengthen the paper. I can see that the authors do not make strong claims about the motor involvement but the experiment data indicates that the execution performance of experts worsens. So, an explanation in terms of forward model and controller/planning interaction would be nice.

In the modeling work, performance degradation is captured by a deterioration of the controller independent the of the forward model; but it could be well the case that some non-trivial interaction between the forward model and the controller is responsible for the performance degradation.

Finally another concern is about the simplicity of the computational model. It would be nice to have authors comment on what aspects of human motor control and/or sensorimotor learning are oversimplified, and whether these may cause significant effects on the final conclusions drawn.

Reviewer #2

The submitted manuscript “Shared mechanisms in the estimation of self-generated actions and the prediction of other's actions by humans”. Forward models (which mimic the processes linking a plant's output to causal inputs) have been a highly influential notion in motor control over the past two decades. Evidence for them has steadily accumulated over the years but less so for understanding how we evaluate and our influenced by the actions of others, despite the natural applicability of the proposed mechanism. Here the authors employ a carefully designed set of experiments and accompanying modelling for this purpose. In brief, verified experts performed open-loop dart throwing and predicted where the darts landed. Between these blocks they watched edited videos of novices throwing darts (or bowling) and estimated the landing (or striking) pattern. For one experimental session, which I'll designate “trainable observations”, experts were told that the novice subjects aimed for the target center and were given the true score following their prediction. In another session, which I'll designate “untrainable observations”, the expert were told that novice subject changed their aim point from trial-to-trial and were not informed about the score. Taken together, these conditions were chosen to provide different influenced on the expert's forward model of the observer's behavior and, consequently, impact the expert's model-dependent actions. The predictions were born out as the expert performance deteriorated in the trainable session with dart but not bowling, and at all during the untrainable session. Since the prediction of the novice's actions improved for both darts and bowling during the “trainable observations” this deterioration was not doe to a general reallocation of resources but a behavior-specific process. The second half of the study involved modelling the self-estimation and behavioral patterns. By considering a variety of models and selectively retaining those which account for the principal results the remaining candidate was indentified to be a forward model with additive noise.

I have only two suggestions.

1. Add some material indicating to how additive or multiplicative noise has been examined before, with respect to relevant sensori-motor systems,

2. Adopting the convenient designations I used above.
